# Strategy for Generating Blinded Evidence for Single-Arm Trials with External Controls Using Expert Review of Home Video

**DOI:** 10.1007/s43441-023-00568-4

**Published:** 2023-08-17

**Authors:** Xinruo Zhang, Katherine Brind’Amour, Kelly E. King, Susan Hartmaier, Katherine Harris, David A. Weinstein, Cynthia J. Girman

**Affiliations:** 1 CERobs Consulting, LLC, Wrightsville Beach, NC USA; 2 Department of Epidemiology, Gillings School of Global Public Health, University of North Carolina at Chapel Hill, Chapel Hill, NC USA; 3 HealthWords Ltd, Augusta, GA USA; 4 Department of Pediatrics, University of Minnesota, Minneapolis, MN USA; 5 University of Connecticut Health Center, Farmington, CT USA

**Keywords:** Rare disease, Home video recording, Developmental milestones, External control, Clinical trial endpoints, Natural history study

## Abstract

**Introduction:**

Neurodegenerative diseases cause developmental delays and loss of milestones in infants and children. However, scalable outcome measures that quantify features meaningful to parents/caregivers (P/CGs) and have regulatory precedence are lacking for assessing the effectiveness of treatments in clinical trials of neurodegenerative disorders. To address this gap, we developed an innovative, blinded strategy for single-arm trials with external controls using expert panel review of home video.

**Method:**

We identified meaningful, observable, and objective developmental milestones from iterative interviews with P/CGs and clinical experts. Subsequently, we standardized video recording procedures and instructions to ensure consistency in how P/CGs solicited each activity. In practice, videos would be graded by an expert panel blinded to treatment. To ensure blinding and quality control, video recordings from interim time points would be randomly interspersed. We conducted a pilot study and a pretest of grading to test feasibility and improve the final strategy.

**Results:**

The five P/CGs participating in the pilot study found the instructions clear, selected activities important and reflective of their children’s abilities, and recordings at-home preferrable to in-clinic assessments. The three grading experts found the videos easy to grade and the milestones clinically meaningful.

**Conclusion:**

Our standardized strategy enables expert panel grading of developmental milestone achievements using at-home recordings, blinded to treatment and post-baseline time points. This rigorous and objective scoring system has broad applicability in various disease contexts, with or without external controls. Moreover, our strategy facilitates flexible, continued data collection and the videos can be archived for future analyses.

**Supplementary Information:**

The online version contains supplementary material available at 10.1007/s43441-023-00568-4.

## Introduction

Neurodegenerative diseases stem from insidious damage to cells and connections in the nervous system that are essential for cognition, behavior, mobility, autonomic functions, and communication [1]. Neurodegenerative diseases presenting in infancy and early childhood are characterized by a lack of attainment of developmental milestones, delays in reaching developmental milestones, and/or loss of acquired developmental milestones after a period of plateau. For example, infants with certain neurodegenerative conditions often experience developmental delays in motor and cognitive functions [2], and may gradually lose or never attain multiple developmental milestones such as crawling, head control, and verbal communication [3].

Given the wide range of affected developmental domains in children with such neurodegenerative disorders, a major barrier to clinical trials is identification of endpoints that are meaningful and important to parents/caregivers (P/CGs) and clinicians. These endpoints must be scalable and feasible to administer over the duration of the clinical trials as children age, and ideally, minimally burdensome for the already-taxed families.

There is no universally accepted endpoint that has regulatory precedence for these conditions, nor one that covers all the impacts of the condition in terms of the way the children feel, function, and survive. We developed an innovative, treatment-masked strategy to assess the achievements of developmental milestones, using expert panel grading of at-home video recordings. Standardization of this process involved minimizing bias and ensuring continuity of assessment over time when remote evaluation was a more feasible option, or if pandemic-related closures necessitated remote evaluation. In the current study, we detailed the development process of our strategy, originally designed for a single-arm clinical trial and parallel natural history study (NHS) in children with neurodegenerative diseases. However, our strategy serves as a versatile framework for future research, adaptable to diverse disease populations and contexts.

## Methods

### Selection of Milestones and Standardization of Video Recording Procedures

**Figure 1.**
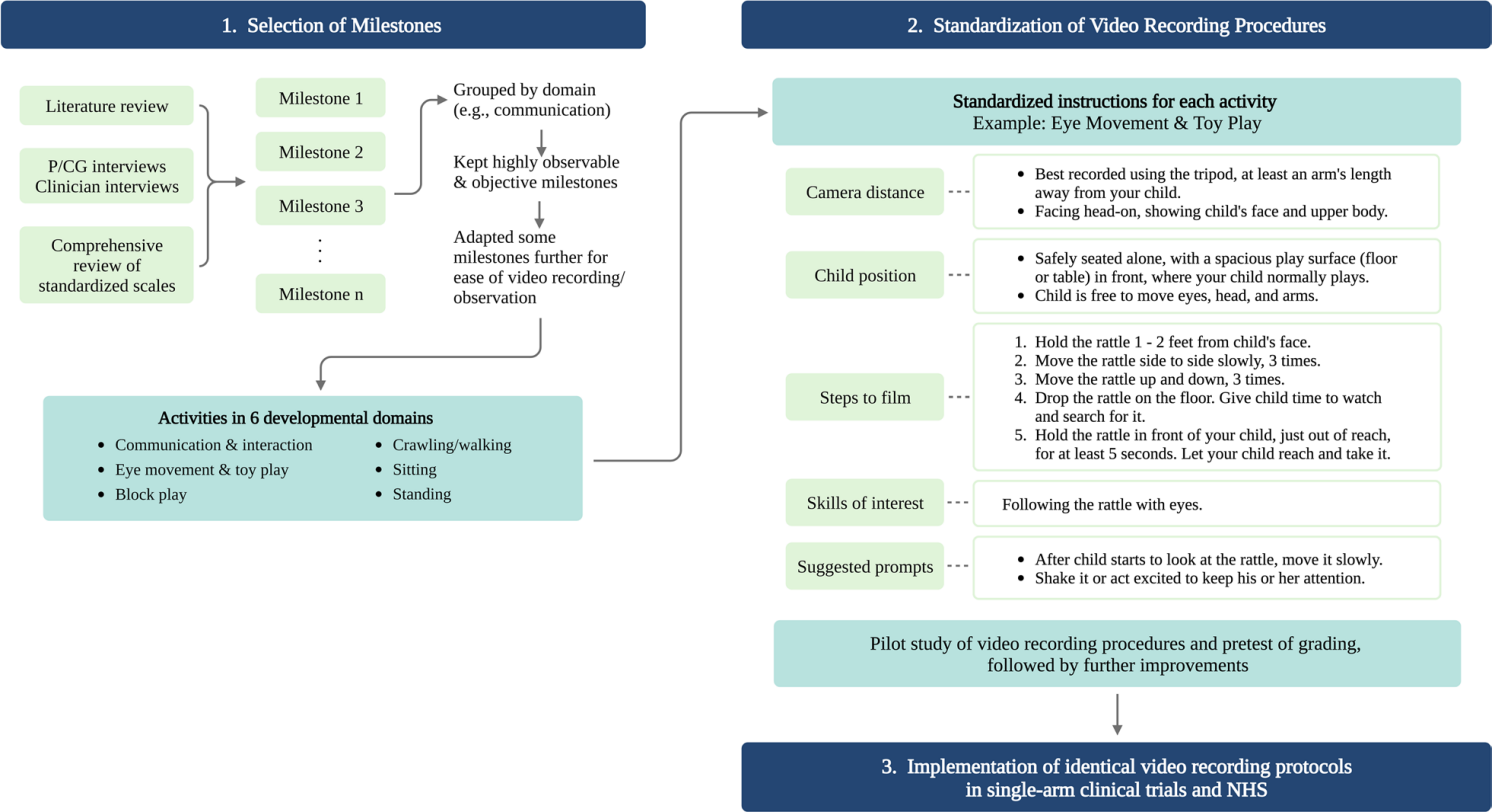
Selection of milestones and standardization of video recording procedures. *NHS* natural history study, *P/CG* parents/caregivers.

At the time of clinical trial planning, there was no standard clinical outcome assessment (COA) for developmental evaluation of children with neurodegenerative conditions of interest. We identified standardized activities to video record through literature review of disease symptoms, iterative interviews with P/CGs and clinical experts, scrutiny of developmental milestones on standardized scales such as the Bayley Scales of Infant and Toddler Development-III (BSID-3) [4] and Vineland Adaptive Behavior Scales-II (VABS-2) [5], and review of child development trackers (Centers for Disease Control and Prevention Developmental Milestones [6] and World Health Organization child growth developmental milestones [7]). The process of selecting and standardizing video-recorded activities is illustrated in Fig. 1.

Developmental milestones were identified that were meaningful to P/CGs and clinical experts and that children with neurodegenerative diseases either never develop or lose as the disease progresses. These were grouped by domain (communication & interaction, eye movement & toy play, block play, crawling/walking, sitting, and standing). This resulted in defined skills across a wide range of ability within a domain that served as the foundation for observing and documenting standardized, discrete activities regardless of age or developmental status. This list was further refined via iterative discussions with clinicians and COA experts to only include milestones that were considered highly observable and objective. They were required to be: (1) easily captured on home video, (2) feasible to observe on video of sufficient quality, (3) easily elicited by P/CGs through basic interactions with their children, (4) objectively gradable by experts in terms of performance, and (5) contributory to experts’ global impression of change (GIC) since baseline. Slight adaptations were further made to certain milestones to make them more appropriate for children with developmental delays due to neurodegenerative disease (e.g., use of a lighted rattle instead of the BSID-3’s unlit ring for eye tracking assessments).

P/CGs prompted and recorded activities by their child in a standardized fashion using a smartphone. The P/CG training materials had a brief manual for the study’s corresponding smartphone app (iTakeControl, RedNucleus, Yardley, PA), which included training videos for each selected activity. A laminated quick reference guide outlining exactly what to do for each activity and providing tips for improving video quality (e.g., avoid filming in front of a window to minimize glare) was also provided (Supplementary Fig. 1). Each of the six skillsets included several basic standardized instructions regarding camera distance, position of child, actions to take during the video, skills being looked for, and examples of how the P/CG could elicit these activities from their children (Fig. 1, Supplementary Fig. 1). P/CGs were allowed to submit up to three videos for each activity, as well as optional videos of their child performing a task not included in the selected activities but deemed important to them. Viewing of training videos was mandatory before video capture or submission for each activity, and training videos could be rewatched at any time. Before the finalization of our strategy, training materials and video recording processes were piloted by P/CGs (see below) and clinical experts. Each submitted video was reviewed by quality control experts using a checklist of 10 criteria (Table 1), although not all of these were considered quality review failure. For instance, excellent-quality videos could be recorded without a tripod, despite encouragement to use one.

**Table 1:** Checklist of 10 quality criteria

Quality criterion	Y/N
1. Was the correct activity assessment submitted?	
2. Was the child positioned properly within the picture frame to allow for activity to be viewed?	
3. Was the distance from the smartphone to the child at least an arm’s length away to allow for activity to be viewed?	
4. Does the recording of the activity appear complete?	
5. Was the video filmed with proper lighting?	
6. Was the video recording in focus, with clear image?	
7. Was the audio recording on the video clear?	
8. Was a tripod used to record the activity?	
9. Was the length of video no more than three minutes?	
10. Did P/CG have difficulty with the app for uploading video?	

### Pilot-Testing of Video Recording Procedures and Pretest of Grading

A small pilot study was conducted in November and December 2020 to understand the feasibility and logistics of recording and submitting high-quality videos and to test interpretability and comprehension of training materials. The pilot study protocol was approved by a central institutional review board (WCG IRB), and interested P/CGs identified by a patient advocacy organization were invited to participate. Eligible participants had the early or late infantile type of a specific neurodegenerative disorder for the clinical trial, and met all of the prespecified inclusion/exclusion criteria for the child (e.g., at least four months old and able to perform a minimum number of pre-specified, simple milestones) and for the P/CG (e.g., 18 years or older, English-speaking, access to a smartphone, willing to video record their child and participate in a follow-up interview). Training materials, a tripod, and required toys (lighted rattle and blocks) were shipped to participants. Within one week of completing their video recordings, P/CGs were scheduled for a one-hour, semi-structured interview conducted over the telephone to collect feedback on the clarity of the training materials and discuss any challenges with the video recording process.

In May 2021, we also conducted a pretest of the expert grading process with three child development expert clinicians to test the feasibility of using video to objectively observe and assess milestones, as well as the appropriateness and practicality of the standardized grading rubric. Before grading, the three experts were trained in a 45-min session that introduced the clinical trial, the video capture pilot study for P/CGs, and the process to follow for viewing videos (in ShareFile) and for grading (temporarily mocked up in SurveyMonkey to mimic the study platform’s planned final grading screens).

These experts were provided with a secure login to a reviewer-specific list of videos in randomized order that was auto-generated using Excel randomization commands. They were instructed to view videos submitted by the P/CGs of the cases in the pilot study in the pre-determined randomized order, side-by-side with the grading rubric, so that they could check off any milestone observed in each video. Items on the grading rubric corresponded to the activities elicited of children by their P/CGs, along with additional milestones that would be commonly observed on the videos. For example, P/CGs were instructed to prompt their children to stand up and walk as independently as possible; expert reviewers could then evaluate the activity shown on the video with corresponding milestones such as “raises self to standing position,” “stands up alone,” “walks alone,” and “walks alone with coordination.” Children could also receive credit for demonstrating skills specifically solicited in other sections of the rubric, such as “turns head to sides,” if observed during a different activity.

Each item on the grading rubric equated to one point, and each item was scored in a dichotomous fashion (achieved/not achieved) based on the rater’s observations of the videos submitted. The total count of milestones achieved (without duplication of points for milestones demonstrated in multiple videos) for each child at each graded time point in a clinical trial would enable derivation of change in the count of milestones achieved longitudinally (vs. baseline). The average change in milestones achieved could be compared between groups (case vs. control in the pilot study; future clinical trial vs. NHS).

After watching all the videos, raters were then asked to view two videos that were obtained from one of the participants over the course of one week and provide their GIC to approximate the study’s planned GIC process. They were given 10 days to complete all grading. Within three to five days of completing grading, a trained interviewer conducted telephone interviews with each expert rater, during which they were probed on the grading process effectiveness and efficiency, the logistics of viewing the video and checking off milestones on the rubric, any difficulties with the overall grading process and instructions, and the milestones selected for scoring. Results from the pilot study and the pretest of expert grading facilitated improvements before the formal design of the strategy was finalized.

## Results

### Pilot Study Results

Five P/CGs consented to participate and were enrolled in the pilot study. All P/CG participants were mothers between 25 and 37 years of age, and the majority were college-educated. There were two healthy control participants, three and 15 months old, and three case participants with the degenerative disease, aged 10, 17, and 43 months. A total of 55 videos were submitted by P/CGs in the pilot study. Some P/CGs submitted more than one video for a single activity. Only the 30 videos submitted by P/CGs of the three cases were graded by the expert panel.

**Figure 2:**
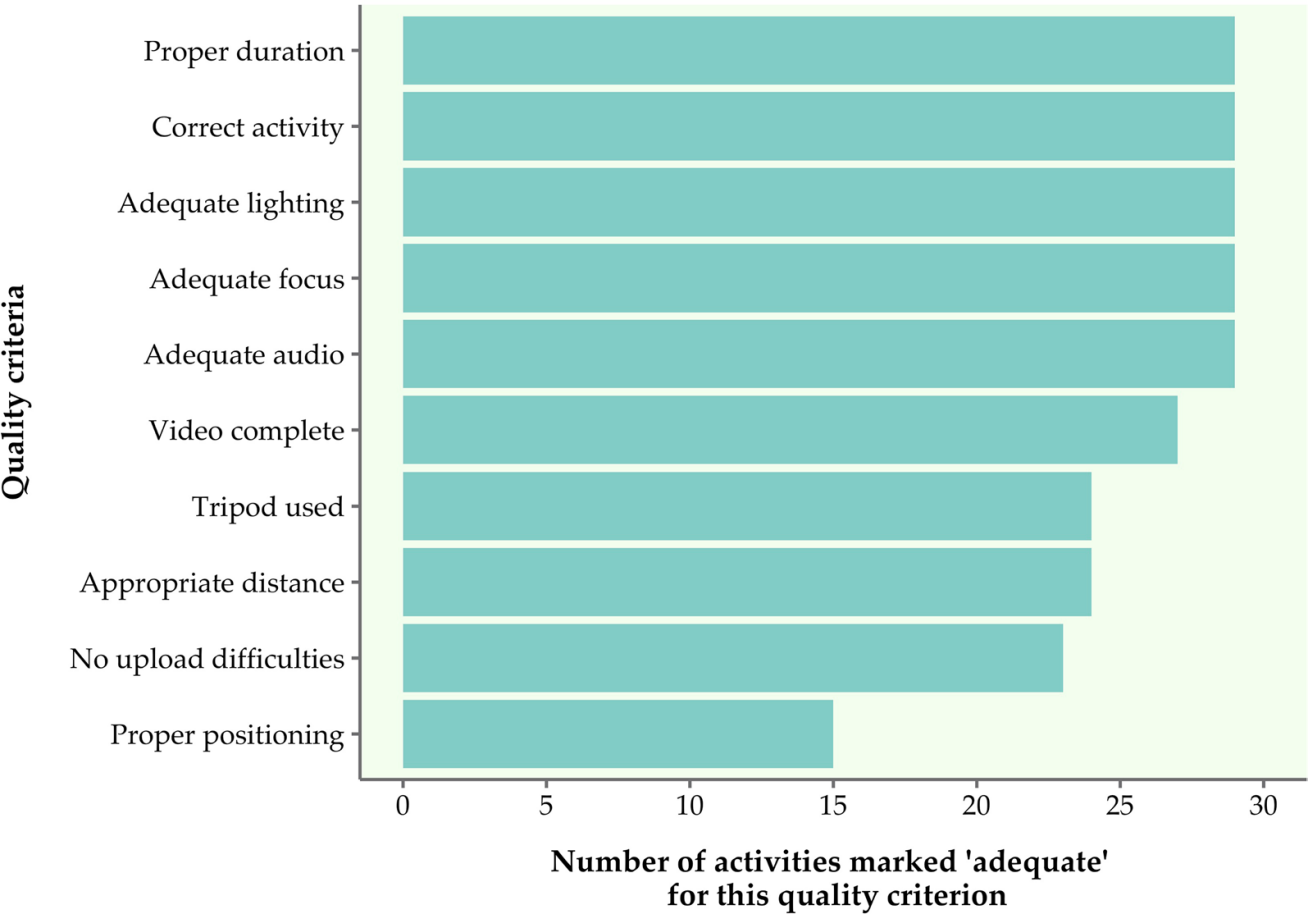
Quality of submitted videos across activities in the pilot study.

All videos were captured and submitted during a single week for each child, and all submitted activities met at least eight of the 10 quality criteria (Table 1). Scores for each quality criterion summarized across all activities for the five P/CGs are illustrated in Fig. 2. One P/CG did not capture the full extent of two activities; she did not put the toy in the camera frame to allow proper evaluation of eye tracking and she also did not offer the child the opportunity to pack/unpack blocks. One P/CG did not use the app to record or upload videos and instead uploaded videos directly from her phone’s video library. Two P/CGs filmed from less than an arm’s length away. Not all activities were filmed using a tripod. Finally, four P/CGs did not have the child appropriately positioned in the frame as instructed in at least one activity (e.g., showing side view instead of frontal view). Despite this, all videos were of sufficient quality to be graded, and the issues that occurred provided the opportunity to improve instructions for the clinical trial training materials.

Scores for each quality criterion were added across all videos submitted by a single P/CG for a single activity. For instance, if a P/CG submitted two videos for ‘sitting’, then this activity was marked ‘adequate’ for the ‘proper duration’ criterion if at least one of the videos was no longer than three minutes.

During interviews, P/CGs reported one week as adequate to complete recordings and said they had no difficulty using their smartphones to record. On average, P/CGs reported spending an hour and half reviewing training materials and videos, which they found clear and easy to follow. In particular, P/CGs found the laminated reference guide extremely useful, referring to it many times before and during filming. P/CGs indicated that all of the selected activities were important to show their child’s developmental milestones and did not recommend any deletions or additions. All of them were pleased to have the opportunity to submit recordings of additional skills. A few P/CGs provided videos of additional skills that were important to them, such as self-feeding. Activities found easiest to record were those where the children were sitting in a chair (i.e., buckled in or not moving around) and/or daily activities (i.e., interacting, smiling, sitting, standing). Activities requiring their child to be mobile (i.e., crawling, walking) were more difficult and often required another person to assist.

There was unanimous response from P/CGs that being able to remotely show their child’s recorded milestone videos to the doctor was preferred over detailed assessments at a clinic visit, as their child would often get fatigued during clinic visits, act differently and display fewer skills at the doctor’s office compared to at-home. Excerpts from P/CG interviews supporting their appreciation of remote assessment included:

- *“It was kind of frustrating for me [at the clinic] … because he was not displaying all the skills that he does at home when he was in that clinical setting.”*
- *“You can see them in their comfortable environment and the way they act a lot more.”*
- *“I think I would prefer having the doctor look at the videos because the child is going to feel safer and more comfortable at his or her own house.”*

### Grading Pretest Results

In the pretest of grading procedures, the three experts took five to eight hours to review 30 videos, with an estimated five to 10 viewings per video. They reported the two-minute video length to be appropriate, and the quality sufficient for scoring purposes. In general, raters found the videos relatively easy to grade and felt that the developmental milestones were clinically meaningful, observable and objective, and appropriate to assess change during a clinical trial. They also felt the milestones could be reasonably captured through home-based videos and were similar to what they would expect in a clinic setting. Raters reported the following video characteristics as barriers to accurate grading: recordings captured from a side profile when full frontal was required, videos not showing if the child was looking at the P/CGs vs. another object, videos cut too short thereby not allowing sufficient time for the child to fully demonstrate a skill (e.g., sitting without support for 30 s), and audio capture that was not quite clear enough to discriminate subtle vocalizations when grading communication-related milestones. Experts also identified the need for clearer definitions of what constituted “tolerates attention,” “nasal” vs. “throaty” sounds, and “persistent reach,” suggesting definitions based on BSID-3 to be added.

In the context of this small pilot study, masking to time point and treatment was not feasible. Nevertheless, experts felt that they would be effectively masked to treatment and likely time, but not to patient identity in a larger study due to the fact that they had graded that patient before. Certain characteristics potentially interfering with limiting recall were identified (e.g., the same P/CG appearing in videos, memorable eyeglasses, or memorable background decor). Overall, raters agreed that being masked to treatment status and randomizing hundreds of videos from dozens of children across various time points, with videos of the same child appearing at least 10 videos apart, would be feasible and effective at minimizing rater recall. Experts agreed that the objective rubric helped eliminate bias when grading the videos.

### Revisions After the Pilot Study and Grading Pretest

Using the pilot and pretest results, we improved the clarity of the activities and milestones, grading rubric, and training materials. Overall, “head control” was separated into its own activity, standing and walking were combined into one video activity (but would be separately graded), crawling and rolling were combined into one video, and the list of milestones in each activity and rubric checklist was reorganized by the order they appear in BSID-3, corresponding to order of difficulty. Moreover, rater feedback also resulted in the addition, deletion, and combination of some milestones. For example, “elevates trunk while prone—shifts weight” was deleted, while “rolls from back to sides” was added. “Undifferentiated throaty sounds” and “undifferentiated nasal sounds” were combined into one milestone. The updated activities and milestones are shown in Supplementary Table 1. Instructions for P/CGs were modified to better enable milestone completion and assessment, i.e., “hold out item for child to reach *for at least 5 s*” (italics indicate added text after pilot and pretest). To further clarify correct steps, professionally recorded sample videos were commissioned for the main trial app for P/CGs to review as ideal examples before attempting their own recordings.

The grading platform was designed to automatically attribute points for directly related milestones when a more advanced milestone was achieved (Table 2). For example, if a rater marked that the child controls his or her head while upright for 15 s, then the child automatically received credit for “controls head while upright, 3 s.” Given the potential for possible recall by certain participant characteristics, P/CG instructions were updated to recommend removal or covering of any identifiers (e.g., child names on furniture or walls) before recording. Anything missed would be blurred by quality control reviewers before a video was released for grading.

**Table 2:** Automatically scored developmental milestones

Milestones granting credit	Milestone automatically credited
Uses 2 words appropriately Uses a two-word utterance Uses a multiple-word utterance Uses different word combinations	Jabbers, jargons, or babbles expressively
Uses 2 words appropriately Uses a two-word utterance Uses a multiple-word utterance Uses different word combinations	2 vowel sounds 2 consonant sounds 1 consonant–vowel combination
Uses a two-word utterance Uses a multiple-word utterance Uses different word combinations	Uses 2 words appropriately
Uses a multiple-word utterance Uses different word combinations	Uses a two-word utterance
Combines word and gesture	Uses gestures
Controls head while upright: 15 s	Controls head while upright: 3 s
Partial thumb opposition Thumb-fingertip grasp	Whole hand grasp
Thumb-fingertip grasp	Partial thumb opposition
Sits with support, 30 s Sits without support, 5 s Sits without support, 30 s	Sits with support, briefly
Sits without support, 5 s Sits without support, 30 s	Sits with support, 30 s
Sits without support, 30 s	Sits without support, 5 s
Rolls from back to stomach	Rolls from back to sides
Stands up, alone Walks alone	Stands alone
Walks, with support Walks alone Walks alone with coordination	Makes stepping movements
Walks, alone Walks, alone with coordination	Walks, with support
Walks, alone with coordination	Walks, alone
Controls head while prone, 90 degrees	Controls head while prone: 45 degrees
Crawls	Elevates trunk while prone: extended arms
Crawls	Crawls on stomach
Makes crawling movements	Crawl position

Lastly, we made changes to the video scripts and the laminated reference guides. For example, the laminated guides were updated to state explicitly that it is acceptable to exceed two minutes of recording, if needed. P/CG instructions were updated to encourage them to submit videos demonstrating a child’s best abilities, even if there were minor interruptions or background noises from other children.

### Final Planned Strategy for the Clinical Trial with Parallel NHS

Identical and standardized at-home video recording instructions are planned for implementation in both the clinical trial and parallel NHS. Videos will be submitted to a secure storage platform via the smartphone app, which will automatically notify P/CGs of their allotted time period (one week or more in duration) for video capture of required activities and send reminders. If they choose not to submit a video for an activity, the reason for not submitting one would be prompted (e.g., “My child is ill”), and study staff will have the option to extend or reschedule the window for video capture if appropriate. Once quality is reviewed and found acceptable, videos will be anonymized and made available in the expert reviewer portal. Site staff could request re-recording of specific activities if needed.

To allow tracking, randomization of videos, and masking to study (trial vs. NHS, or treatment) and time (e.g., baseline vs. 12 months), each video in the trial and NHS will be assigned a unique sequence number. Expert panel grading sessions will be delayed until study accrual enables separating videos of the same child by a minimum of 10 videos to minimize recall. Expert raters will have the flexibility to view each child’s video recordings as many times as they prefer during their review. In addition to checking off milestones observed, experts will also view side-by-side video recordings at baseline and a follow-up time (e.g., 24 months) to assess GIC using a nine-point scale ranging from extremely deteriorated to extremely improved.

**Figure 3.**
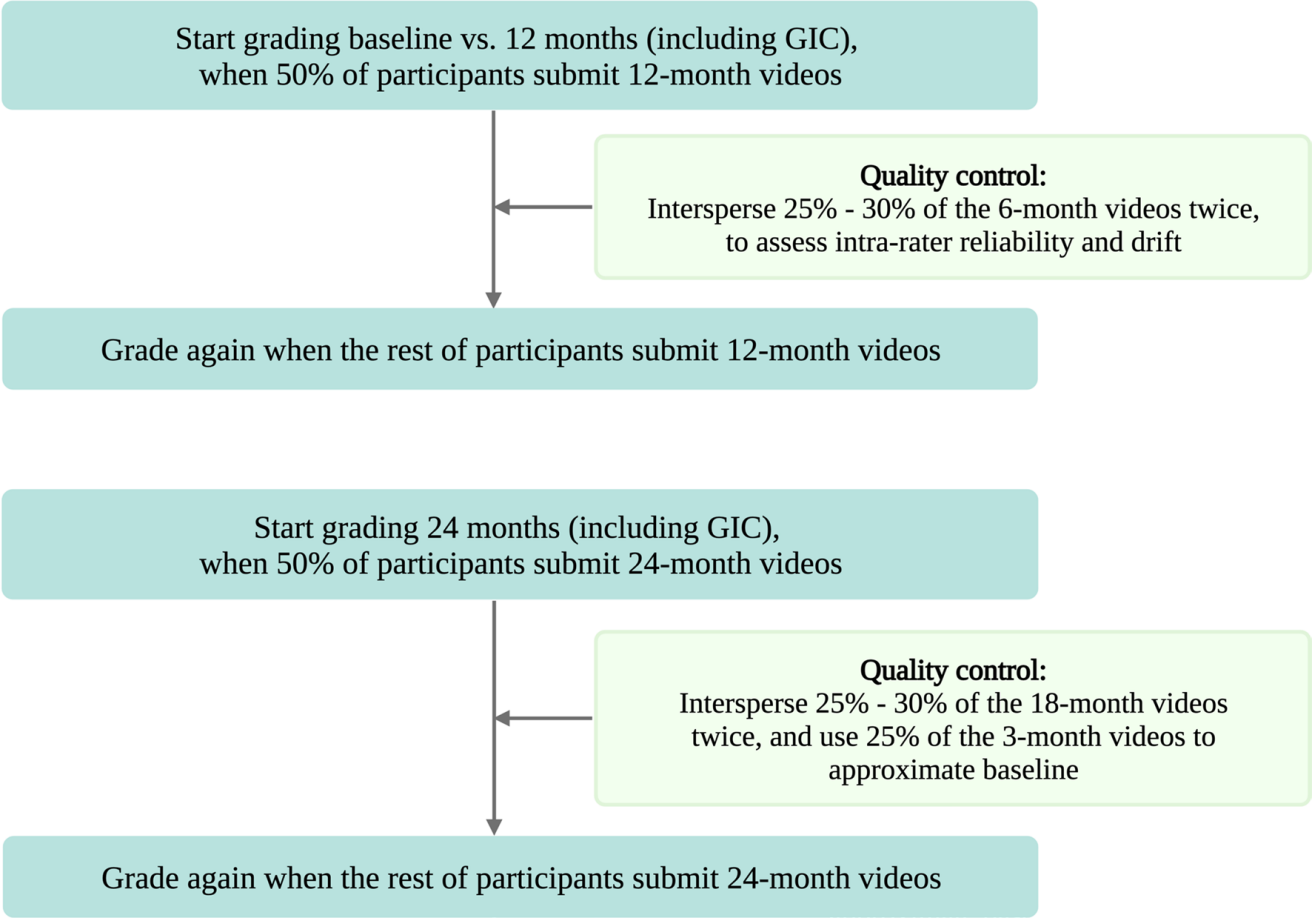
Video grading and quality control plan.

For quality control, plans are to randomly intersperse 25% to 30% of videos from an interim timepoint twice to allow assessment of intra-rater reliability and to monitor raters for drift in grading over time (Fig. 3). Instead of grading baseline videos twice, 25% of the videos from early assessments (e.g., three or six months post-baseline) will be used to approximate baseline. Grading will not begin until 50% of the participants submit their 12-month videos. A second round of grading will commence upon receipt of the 12-month recordings from the remaining participants. This process is to be repeated for the 24-month time point, with 18-month videos used for quality control. These quality control and masking procedures are planned for both the clinical trial and NHS to allow blinding for study (i.e., treatment).

The planned enrollment size was 14 for the clinical trial and 10 for the NHS. Video collection time points were 0, 3, 6, 9, 12, 18, and 24 months. *GIC, global impression of change.*

## Discussion

We have developed an innovative strategy for assessing developmental milestone achievements in children with rare neurodegenerative diseases. The grading of at-home videos from a single-arm clinical trial and parallel NHS exemplifies one practical implementation of our strategy. To our knowledge, this is the first planned use of natural history patients to blind reviewers grading videos in a clinical trial, aiming to evaluate treatment effects and minimize potential bias from knowledge of interventions. Furthermore, our strategy holds potential for adaptation and implementation in studies conducted in other disease populations, with or without external controls, where developmental milestones are integral to evaluating treatment outcomes.

In January 2019, the Food and Drug Administration (FDA) released a draft guidance on rare diseases emphasizing the need to “standardize the collection and handling of data to ensure quality and interpretability” and that “standardized operating procedures and quality assurance and quality control are essential” [8]. Given that research of rare diseases is often conducted at multiple sites due to disease rarity, standardization of data collection and handling is crucial. By selecting only highly observable and objective milestones and standardizing activity solicitation methods, video capture procedures, camera frame for each activity, and expert grading, numerous barriers to generating high-quality data from video recordings are removed. In the draft guidance, the FDA also supports endpoint selection that involves “the aspects of disease that are meaningful to the patient (and caregiver) and that could be assessed to evaluate the drug’s effectiveness” [8]. In the pilot study interviews, P/CGs expressed that the activities were meaningful indicators of their child’s developmental course. Expert interviews also repeatedly supported the importance of these disease aspects to clinicians, including their potential to indicate meaningful change in disease status due to treatment or disease progression. While the feedback received from both P/CGs and experts provides qualitative validation for our strategy, further research is needed to quantitatively evaluate test–retest reliability, construct validity, and responsiveness in a future trial.

While there is minimal, if any, precedence for home videography as the basis for assessing effectiveness to gain approval for products, there is a great deal of precedence in the use of photographic and imaging techniques in drug development. This includes products using automated or expert measurement or grading of 2-D and 3-D photography or imaging, such as static head or bald spot images in alopecia [9, 10], skin images in dermatology [11, 12], tumor assessments in oncology [13] and prostate size in urology [14]. Remote video assessments are also increasingly utilized in clinical care, diagnostics, and ongoing assessment, even in the case of developmental disorders affecting numerous domains [15, 16]. Our strategy is innovative due to its heavy involvement of P/CGs and clinical experts, iterative nature of the design process, and customization specific to our disease of interest. However, these procedures are easily adaptable to other conditions and diseases where developmental or functional status is difficult to assess using a single outcome. Videos allow the assessment of objective dynamic parameters that static pictures do not easily allow, including language skills or time-dependent motor functions such as sitting for five seconds without support. Furthermore, videos can be saved and re-analyzed by blinded experts after more evidence/videos accumulate, which can be useful in studies of rare diseases that accrue patient data slowly; this is particularly meaningful in the field of rare diseases, where studies are limited by the paucity of participants.

Remote video assessments also have several disease-agnostic benefits for clinical trials and rare disease follow-up in neurodiverse populations. They offer increased flexibility for continued data collection in circumstances of interrupted visit schedules or decentralized trials aiming to minimize site visits [17]. In light of the recent COVID-19 pandemic, the ability to record videos at-home greatly reduces burdens of travel, which can be particularly challenging for P/CGs with children affected by neurodegenerative conditions and significant medical needs. Video recording activities at-home, on the participant and P/CG’s schedule, allows for data collection in a familiar environment by a familiar caregiver when the participant is most alert and feeling their best. This approach reduces stress on the child, minimizing the risk of white-coat syndrome and offering an opportunity to improve data robustness for pediatric participants. P/CGs reported that home video recordings more accurately capture their children’s abilities than in-clinic visits because of their children’s increased comfort level in their home environment and the flexibility to record at the most convenient time, rather than being constrained to a specific clinic appointment time when children may be hungry, afraid, struggling to cooperate, jet-lagged, or generally fatigued.

Our video review and grading procedures allow the generation of rigorous, objective scoring by masking raters to study/treatment and to post-baseline time since treatment in the trial or since enrollment in the NHS. Expert feedback suggested that the masking efforts planned for the study were the most appropriate and effective approach to masking for such video review. Natural aging and change in appearance of children over time may further limit the ability to recall status of specific children over time and may further enhance the ability to mask to child identity. Nonetheless, it is worth noting that the success of randomized order and effective masking hinges on the availability of a large number of videos from both the trial and an NHS, which may be challenging in the context of rare disease if video recordings are captured infrequently.

Our strategy can also be applied to studies outside of the United States. Even though not tested in the pilot study, local developmental experts or study staff could provide notes for activities documenting culture-specific gestures, vocalizations, and words in videos collected from families not speaking English, so that a centralized expert panel could review.

## Conclusions

Incorporating input from P/CGs and clinicians, we designed a standardized COA strategy using at-home video recordings that can be tailored to other diseases. Meaningful outcomes can be gleaned from expert grading of standardized at-home video recordings to assess the effectiveness of therapy. By implementing such procedures in both a clinical trial and a parallel NHS, treatment can be masked even for a single-arm, open-label clinical trial.

### Supplementary Information

Below is the link to the electronic supplementary material.

Supplementary file1 (DOCX 21 KB)

Supplementary file2 (PDF 160 KB)

## Data Availability

Not applicable.
