# Supplementary material for: Strategy for Generating Blinded Evidence for Single-Arm Trials with External Controls Using Expert Review of Home Video
Source: Ther Innov Regul Sci. 2023 Aug 17;57(6):1304–13. doi: 10.1007/s43441-023-00568-4 (PMC10579152; doi:10.1007/s43441-023-00568-4)
Supplement: Supplementary file 1 — Supplementary file1 (DOCX 21 KB) [file 43441_2023_568_MOESM1_ESM.docx]

**Supplementary Table 1**. The final activities and milestones after the pilot study and the pretest of grading

| **Activity 1: Communication and Interaction** |
| --- |
| Regards person momentarily |
| Tolerates attention |
| Respond to a person’s voice |
| Undifferentiated throaty or nasal sounds |
| Social smile |
| Social vocalizing or laughing |
| 2 vowel sounds |
| 2 constant sounds |
| Uses gestures |
| 1 consonant-vowel combination |
| Jabbers, jargons, or babbles expressively |
| Uses 2 words appropriately |
| Combines word and gesture |
| Answers yes or no verbally in response to questions |
| Uses a two-word utterance |
| Uses multiple-word utterances |
| Uses different word combinations |
| **Activity 2: Eye Movement and Attention** |
| Regards object for 3 seconds |
| Becomes excited in anticipation |
| Shifts attention |
| Prefers novel object |
| Eyes follow rattle horizontally |
| Eyes follow rattle vertically |
| Head follows rattle |
| Interrupts activity |
| **Activity 3: Head Control** |
| Controls head while upright: lifts head |
| Controls head while upright: 3 seconds |
| Turns head to sides |
| Controls head while upright: 15 seconds |
| Holds head in midline |
| Controls head while prone: 45 degrees |
| Controls head while prone: 90 degrees |
| **Activity 4: Block and Toy Play** |
| Explores object |
| Carries object to mouth |
| Persistent reach |
| Bangs in play |
| Searches for fallen object |
| Takes blocks out of container |
| Puts 1 block in the container |
| Attempts to bring hand to mouth |
| Retains rattle |
| Keeps hands open |
| Rotates wrist |
| Reaches for rattle |
| Touches rattle |
| Whole hand grasp |
| Reaches unilaterally |
| Partial thumb opposition |
| Transfers block or object |
| Thumb-fingertip grasp |
| Brings blocks (or other items) to midline |
| Stacks 2 blocks |
| Stacks 6 blocks |
| **Activity 5: Sitting** |
| Sits with support, briefly |
| Sits with support, 30 seconds |
| Sits without support, 5 seconds |
| Pulls up to sit |
| Sits without support, 30 seconds |
| **Activity 6: Crawling and Rolling** |
| Makes crawling movements |
| Elevates trunk while prone: Elbows and forearms |
| Rolls from back to sides |
| Elevates trunk while prone: Extended arms |
| Rolls from back to stomach |
| Rolls from stomach to back |
| Crawls on stomach |
| Crawl position |
| Moves from sitting to hands and knees |
| Crawl movement |
| **Activity 7: Standing and Walking** |
| Makes stepping movements |
| Supports weight |
| Raises self to standing position |
| Walks, with support |
| Stands alone |
| Stands up alone |
| Walks, alone |
| Walks, alone with coordination |
| Stands up, mature |
