# Supplementary material for: Strategy for Generating Blinded Evidence for Single-Arm Trials with External Controls Using Expert Review of Home Video
Source: Ther Innov Regul Sci. 2023 Aug 17;57(6):1304–13. doi: 10.1007/s43441-023-00568-4 (PMC10579152; doi:10.1007/s43441-023-00568-4)
Supplement: Supplementary file 2 — Supplementary file2 (PDF 160 KB) [file 43441_2023_568_MOESM2_ESM.pdf]

1

Communication and Interaction

PURPOSE: *Show your child's ability to engage with you.*

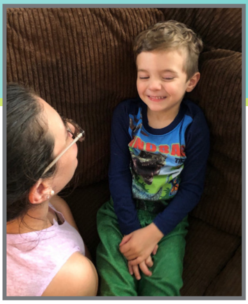

VIDEO SETUP:

- **POSITION:** Seated or reclined
- **SHOWING:** Child's face and upper body (shoulders and arms), and your head when you interact
- **HOW TO FILM:** About an arm's length away or more

SPECIAL NOTES: *It is best to record with the camera on the tripod, at least an arm's length from your child. If you get help from another adult, use the same adult each time you record this activity.*

INSTRUCTIONS:
Try to get your child to smile, laugh, babble, gesture, or talk by asking questions, tickling, kissing, etc.

Skills we are looking for:	**TRY THIS!**
▪ SMILING<br>▪ LAUGHING	Tickle, smile, make silly faces<br>Caress or squeeze gently<br>Have your child's brother or sister help
▪ LOOKING AT YOU<br>▪ REACTING TO YOU OR FAMILY MEMBER	Look your child in the eyes<br>Talk, sing, kiss, smile, make silly faces,<br>Walk away for a moment<br>Touch his or her face
▪ COOING, BABBLING, GURGLING, JABBERING<br>▪ DIFFERENT NOISES (OOOH, AAHH, BABA, MAMAMA)<br>▪ WORDS (UH-OH, NO, BYE-BYE, DADA, BALL, ETC.)<br>▪ ANSWERING QUESTIONS<br>▪ CONVERSATION	Make different noises<br>Ask 'yes or no' questions ("Do you want this toy?") and wait for a response<br>Ask conversational questions ("What are you thinking about?")<br>Kiss or snuggle
▪ GESTURES LIKE WAVING, CLAPPING, SHAKING OF HEAD, OR BLOWING KISSES	Wave at your child, blow kisses, play peekaboo or other games<br>Use sign language
